# Involvement of Trigeminal Transition Zone and Laminated Subnucleus Caudalis in Masseter Muscle Hypersensitivity Associated with Tooth Inflammation

**DOI:** 10.1371/journal.pone.0109168

**Published:** 2014-10-03

**Authors:** Kohei Shimizu, Kunihito Matsumoto, Noboru Noma, Shingo Matsuura, Kinuyo Ohara, Hiroki Komiya, Tetsuro Watase, Bunnai Ogiso, Yoshiyuki Tsuboi, Masamichi Shinoda, Keisuke Hatori, Yuka Nakaya, Koichi Iwata

**Affiliations:** 1 Department of Endodontics, Nihon University School of Dentistry, Tokyo, Japan; 2 Department of Radiology, Nihon University School of Dentistry, Tokyo, Japan; 3 Divisions of Advanced Dental Treatment, Dental Research Center, Nihon University School of Dentistry, Tokyo, Japan; 4 Department of Oral Diagnosis, Nihon University School of Dentistry, Tokyo, Japan; 5 Divisions of Clinical Research, Dental Research Center, Nihon University School of Dentistry, Tokyo, Japan; 6 Department of Physiology, Nihon University School of Dentistry, Tokyo, Japan; 7 Division of Functional Morphology, Dental Research Center, Nihon University School of Dentistry, Tokyo, Japan; 8 Division of Applied System Neuroscience Advanced Medical Research Center, Nihon University Graduate School of Medical Science, Tokyo, Japan; University of Texas at Dallas, United States of America

## Abstract

A rat model of pulpitis/periapical periodontitis was used to study mechanisms underlying extraterritorial enhancement of masseter response associated with tooth inflammation. Periapical bone loss gradually increased and peaked at 6 weeks after complete Freund’s adjuvant (CFA) application to the upper molar tooth pulp (M1). On day 3, the number of Fos-immunoreactive (IR) cells was significantly larger in M1 CFA rats compared with M1 vehicle (veh) rats in the trigeminal subnucleus interpolaris/caudalis transition zone (Vi/Vc). The number of Fos-IR cells was significantly larger in M1 CFA and masseter (Mass) capsaicin applied (M1 CFA/Mass cap) rats compared with M1 veh/Mass veh rats in the contralateral Vc and Vi/Vc. The number of phosphorylated extracellular signal-regulated kinase (pERK)-IR cells was significantly larger in M1 CFA/Mass cap and M1 veh/Mass cap rats compared to Mass-vehicle applied rats with M1 vehicle or CFA in the Vi/Vc. Pulpal CFA application caused significant increase in the number of Fos-IR cells in the Vi/Vc but not Vc on week 6. The number of pERK-IR cells was significantly lager in the rats with capsaicin application to the Mass compared to Mass-vehicle treated rats after pulpal CFA- or vehicle-application. However, capsaicin application to the Mass did not further affect the number of Fos-IR cells in the Vi/Vc in pulpal CFA-applied rats. The digastric electromyographic (d-EMG) activity after Mass-capsaicin application was significantly increased on day 3 and lasted longer at 6 weeks after pulpal CFA application, and these increase and duration were significantly attenuated by i.t. PD98059, a MEK1 inhibitor. These findings suggest that Vi/Vc and Vc neuronal excitation is involved in the facilitation of extraterritorial hyperalgesia for Mass primed with periapical periodontitis or acute pulpal-inflammation. Furthermore, phosphorylation of ERK in the Vi/Vc and Vc play pivotal roles in masseter hyperalgesia after pulpitis or periapical periodontitis.

## Introduction

Orofacial persistent pain following trigeminal nerve injury or orofacial inflammation is known to cause various motor as well as sensory disorders in the orofacial regions such as mastication or swallowing dysfunction [1]. Orofacial dysesthesia is known as the secondary hyperalgesia often associated with pulpitis and/or periapical periodontitis [2], suggesting that oral and craniofacial organs are target regions for ectopic pain following intraoral inflammation. Furthermore, it has been reported that the chronic orofacial pain associated with pulpitis and/or periapical periodontitis is one of the most frequent referred pain in the orofacial region, and pulpitis or periapical periodontitis is also known to be involved in the referred pain in various intraoral structures [3], [4]. Referred pain in non-inflamed orofacial areas associated with pulpitis or periapical periodontitis is often accounted for misdiagnosis or inappropriate clinical treatment. Thus, it is very important to understand the mechanisms underlying orofacial-referred pain associated with pulpitis or periapical periodontitis to develop appropriate diagnosis and treatment of these patients.

The spinal trigeminal subnucleus caudalis (Vc) has been known as a key nucleus to relay tooth pulp afferent inputs to the higher central nervous system, and a large number of nociceptive neurons in the Vc are activated by orofacial noxious stimulation as well as tooth pulp stimulation [5], [6]. Electrical stimulation of the tooth pulp produces Fos protein expression in many neurons in the transition zone between the subnucleus interpolaris (Vi) and Vc (Vi/Vc) and the caudal Vc and upper cervical cord [5]. These two areas are thought to play differential roles in processing noxious information from the tooth pulp as well as orofacial skin or muscles [7], [8]. It has been reported that Vi/Vc and Vc have a role to process masseter muscle pain [9]. Furthermore, many neurons in these two areas are also known to receive noxious as well as non-noxious inputs from the orofacial regions [7], [10]. Therefore, it is highly possible that nociceptive neurons in the Vi/Vc and Vc enhance their firings following pulpitis or periapical periodontitis and play a role in orofacial cutaneous and/or Mass nociception as well as processing tooth pulp or periapical inputs. We hypothesized therefore that Vi/Vc and Vc neurons are involved in masseter muscle hypersensitivity associated with tooth pulp and periapical inflammation.

Extracellular signal-regulated protein kinase (ERK) is a member of mitogen-activated protein kinase family that can be activated by calcium influx within 10 min after noxious stimulation [11]. Phosphorylated ERK-immunoreactive (pERK-IR) cells are known to be distributed in Vc with somatotopic organization, and the number of pERK-IR cells increased in an intensity-dependent manner in the Vc [12]. Previous studies indicated that C-fiber but not A-fiber stimulation accelerates ERK phosphorylation in dorsal root ganglion and spinal dorsal horn (SDH) neurons [13]. These findings indicate that ERK phosphorylation in Vc neurons could be a reliable indicator of neuronal activation following noxious stimulation of the orofacial regions.

Fos-IR cells have been widely used as a marker of neuronal activation in the SDH following noxious stimulation [14]. Fos expression could be detected 0.5–1 hour after noxious stimulation and lasts for 2–3 hours after stimulation. Recently, we have also reported that many pERK-IR cells show Fos-IR in Vc neurons following noxious mechanical stimulation of the periodontal tissues [15], suggesting that ERK phosphorylation is followed by Fos protein expression after noxious stimulation.

To clarify the central mechanisms underlying Mass hypersensitivity associated with pulpitis or periapical periodontitis following CFA application to the tooth pulp, we studied ERK phosphorylation and Fos expression in Vi/Vc and Vc neurons in tooth pulp- or periapical inflamed rats.

## Materials and Methods

This study was approved by the Animal Experimentation Committee at the Nihon University (Animal protocol AP10D001). All surgery and animal care were conducted in accordance with the National Institutes of Health Guide for the Care and Use of Laboratory Animals and the guidelines for Institutional Animal Care, and the guidelines of the International Association for the Study of Pain [16]. Male Sprague-Dawley rats (n = 152) weighing 250–450 g were used in this study. The rats were housed under 12 h light/dark cycle conditions and had free access to food and water except during the test period. To minimize animal suffering, the number of animals used was based on the minimum required for statistically valid results. Immunohistochemical and Electrophysiological analysis were carried out in a blind manner.

### Pulpal or periapical inflammation

Rats were lightly anesthetized with 2% isoflurane (Mylan, Canonsburg, PA), then deeply anesthetized with intraperitoneal (i.p.) application of sodium pentobarbital (50 mg/kg; Schering Plough, Whitehouse Station, NJ), and placed on a warm mat (37°C) in a dorsal recumbent position. To allow for application of CFA (n = 4, Sigma-Aldrich, 50% CFA was diluted in saline) or vehicle (n = 4, isotonic physiological saline) to the right maxillary first molar (M1) tooth pulp, the rats’ mouth was gently opened and the dental pulp was exposed by means of low-speed dental drill with a round tungsten carbide bur (No. 1-4: JOTA, Tokyo) under water cooling. CFA or vehicle was applied to the tooth pulp by a small piece of dental paper point (diameter, 0.15 mm; length, 1.5 mm; PIERCE ABSORBENT POINTS, #15) soaked with CFA or vehicle. After CFA or vehicle application with dental points, the cavity was tightly sealed with dental cement (GC Fuji I, Tokyo). The bone loss around the tooth apex was evaluated using in vivo Micro X-ray CT System R_mCT (R_mCT, Rigaku, Tokyo). There were no any abnormal animal behaviors such as grooming, locomotion and feeding. The rats did not lose weight during experimental period.

The sagittal CT images for tooth apex were obtained at 300 µm intervals, and then the bone loss around tooth apex was drawn using Neurolucida (Micro-Brightfield Inc., Colchester, VT). The drawn images were integrated and 3D image of the periapical bone loss was constructed. The change in the volume was calculated by the Neuroexplore software (Micro-Brightfield Inc., Colchester, VT). Furthermore, on day 3 or week 6 after CFA application to upper molar tooth pulp, rats were anesthetized with sodium pentobarbital (80 mg/kg, i.p.) and perfused with 4% paraformaldehyde. Maxillar bone and M1 were removed and decalcified overnight and 15 mm serial sections were cut. Then sections were stained with hematoxylin and eosin and coverslipped.

### pERK and Fos immunohistochemistries

For double immunofluorescence histochemistry for pERK and NeuN, sections were rinsed in PBS, 10% normal goat serum in PBS for 1 h, and then incubated in rabbit antiphospho-p44/42 MAP Kinase Antibody (1∶200, Cell Signaling Technology) for 72 h at room temperature. Then the sections were incubated in mouse anti-neuronal nuclei monoclonal Antibody (1∶500, Chemicon, Temicula, CA) for 2 h at room temperature. Next, the sections were incubated in Alexa Fluor 488 anti-rabbit IgG (1:200 in 0.01 M PBS; Invitrogen, Paisley) and in Alexa Fluor 568 anti-mouse IgG (1∶200 in 0.01 M PBS; Invitrogen) for 2 h at room temperature. After rinsing with 0.01 M PBS, sections were coverslipped in mounting medium (Thermo Fisher Scientific, Fremont, CA) and examined under a fluorescence microscope and analyzed using a BZ-9000 system (Keyence, Osaka).

At 3 days and 6 weeks after CFA or vehicle application to M1 (n = 6 each), rats were deeply anesthetized with 2% isoflurane, and a 26 G needle with a Hamilton syringe was inserted into the ipsilateral Mass. After 30 min rest period, capsaicin was applied to the ipsilateral Mass, and at 5 minutes after capsaicin application, rats were transcardially perfused with isotonic saline followed by a fixative containing 4% paraformaldehyde in 0.1 M phosphate buffer (pH 7.4). The whole brain including medulla and upper cervical cord was removed and post-fixed in the same fixative for 3 days at 4°C. The tissues were then transferred to 20% sucrose (w/v) in 0.01 M phosphate-buffered saline (PBS) for several days for cryoprotection. Thirty-micron-thick sections were cut with a freezing microtome, and every fourth section was collected in PBS. Free-floating tissue sections were washed in PBS and rinsed in 1% hydrogen peroxide with 0.75% Triton X-100 (Sigma, St. Louis, MO) in PBS for 1 hour. After washing in PBS, the sections were incubated in 10% normal goat serum in PBS, and then incubated in polyclonal rabbit anti-Fos antibody (c-Fos ab-5, 1∶5,000 dilution; Oncogene, Cambridge, MA) for overnight at 4°C. Next, the sections were incubated in biotinylated goat anti-rabbit IgG (1∶600; Vector Labs, Burlingame, CA) for 2 h at room temperature. After washing in PBS, the sections were incubated in peroxidase-conjugated avidin-biotin complex (1∶100; ABC, Vector Labs) for 2 h at room temperature. After washing in 0.05 M Tris Buffer (TB), the sections were incubated in 0.035% 3,3-diaminobenzidine-tetra HCl (DAB, Sigma, St. Louis, MO), 0.2% nickel ammonium sulfate and 0.05% peroxide in 0.05 M TB (pH 7.4). After washing in PBS and incubated in 10% normal horse serum in PBS, the sections reacted with Fos antibody were incubated in mouse anti-phospho-p44/42 MAP Kinase Antibody (pERK, 1 1000, Cell Signaling Technology) for 72 h at 4°C. The following immunohistochemical procedures were similar to those of c-Fos immunohistochemistry. The reaction products of biotinylated horse anti-mouse antiserum and avidin-conjugated horseradish peroxidase were visualized with 0.035% DAB and 0.05% hydrogen peroxide without nickel.

The sections were washed in PBS, serially mounted on gelatin-coated slides, dehydrated in alcohols and cover slipped. The pERK-IR, Fos protein-IR and double-labeled neurons were drawn under the light microscope using Neurolucida drawing tube. The neurons stained with DAB as brown in the nuclei and cytoplasm were determined as pERK-IR cells. The neurons stained with DAB as black in the nuclei were determined as Fos-IR cells. The number of pERK-IR, Fos protein-IR neurons was counted from every 8th section. The total number of IR neurons from 3 of every 8th section was calculated, and the mean number of those cells (3 sections/rat) was obtained from each animal.

### Digastric electromyographic recording after capsaicin application into the Mass

On day 3 or week 6 after CFA or vehicle application to the M1 tooth pulp (n = 6 each), rats were anesthetized with 2% isoflurane and a pair of bipolar wire electrodes (enamel-coated stainless steel wire, inter-polar distance: 5 mm) was inserted into the digastric muscle on the side ipsilateral to the tooth. After that, a 26G needle connected with a Hamilton syringe was gently inserted into the ipsilateral Mass.

The digastric electromyographic (d-EMG) activity was continuously monitored before, during and after the application of capsaicin, dissolved in 100% ethanol and 7% Tween 80 in saline (3 µM, 100 µl), into the Mass. Before capsaicin application, baseline of d-EMG activity was monitored for 20 min, and d-EMG activity was continuously monitored for an additional 20 min. Then, the d-EMG activity was amplified, rectified and integrated, and the area under the curve of d-EMG activity was calculated by the Spike 2 software (CED, Cambridge). The area under the curve of d-EMG activity was measured for every 1 min before and after capsaicin application, and mean d-EMG activity was calculated in each time period. The calculated d-EMG value more than 2 standard deviation of the baseline after capsaicin application was selected and evaluated.

### PD98059 administration

Rats were anesthetized with 2% isoflurane and placed in a stereotaxic apparatus. After a midline skin incision, an opening was made in the caudal part of the skull with a dental drill to insert intrathecally (i.t.) a soft polyethylene tube (PE45, ID, 0.58 mm; OD, 0.96 mm; Natsume, Tokyo, Japan) [17]. The tube was connected to a mini-osmotic pump (Alzet model 2001, Alzet, Cupertino, CA, USA; total volume, 200 µL) filled with the drug and embedded subcutaneously in the dorsal portion of the body. Thus, the MEK1 inhibitor PD98059 was intrathecally applied for 7 days (1.0 µL/h). The dosage and duration of the drug (0.1 µg/µL) for pump infusion was chosen primarily based on previous reports [18]–[21]. For the rats with periapical periodontitis, CFA was applied into the M1 at 35 days before PD98059 or saline administration, and then capsaicin was applied to the Mass on 7 days after PD98059 administration. For the rats with pulpitis, CFA was applied into the M1 at 4 days after PD98059 or saline administration, and then capsaicin was applied into the Mass at 3 days after CFA application. The d-EMG activity was continuously monitored before and after capsaicin application (n = 6 each).

For pERK and Fos immunohistochemistries, capsaicin was applied into the Mass in CFA-treated rats on day 7 after PD98059 or saline administration (n = 6 each). At 5 minutes after capsaicin application, the rats were perfused, and the pERK and Fos immunohistochemistries were performed.

All subsequent d-EMG recording and staining steps and counting analysis were the same as described above. All d-EMG recording and immunohistochemical experiments were conducted on animals without any obvious neurological deficits. Also, the Alzet pump was removed at the end of each experiment, and the amount of the drug remaining in the pump was checked. If there was still residual drug in the pump, the data from that rat were excluded from the final analysis.

### Statistical analysis

Data were expressed as mean ± SEM. Statistical analyses were performed by Student’s t-test, one-way or two-way repeated-measures analysis of variance (ANOVA) followed by Turkey or Dunnet test appropriately. A value of p<0.05 was considered as significant.

## Results

### 1. The effect of CFA on tooth and periapical tissues

**Figure 1:**
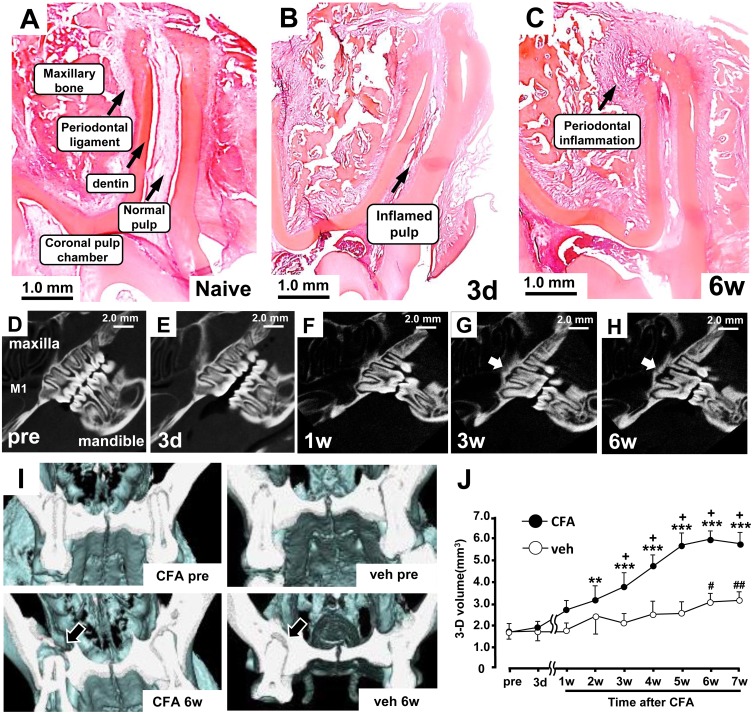
The change in the apical periapical tissues after CFA application to M1 tooth pulp. A, B and C: Photomicrographs of periapical tissues of the naive rats (A) and the rats on day 3 (B) and week 6 (C) after CFA application to the pulp. D–H: Lateral views of 3D X-lay photographs of periapical tissues of pre-CFA application (D), on day 3 (E), on 1 week (F), 3 week (G) and 6 week (H) after CFA application. I: Sagittal views of X-lay micro-CT images of the periapical tissues before and at 6 week after CFA or vehicle application to the pulp. J: Time course change in the size of the bone loss at the periapical tissues after CFA application to the M1 pulp (n = 4 each). Solid arrows in A-C indicate detail tooth and periapical structures. White arrows in G and H, and black arrow in I indicate periapical bone loss. veh: vehicle in this and following figures. *: Pre vs. post (CFA), #: Pre vs. post (veh), +: CFA vs. veh, +, #: p<0.05, ##, **: p<0.01, ***: p<0.001.

The application of CFA to the M1 tooth pulp induced inflammation in the tooth pulp on day 3 and in the periapical tissues on week 6 (Fig. 1). The detail tooth structures of M1 were indicated in Fig. 1A–C. In naive rats or the rats with CFA application to the M1 tooth pulp on day 3, periapical bone loss was not observed (Fig. 1A, B), indicating that the CFA-induced inflammation is limited within the pulp or at the apex region of the tooth. The periapical bone loss was clearly detected at 3 weeks after CFA application to the pulp, as shown by formation of granuloma in the periapical periodontitis (Fig. 1C–I). The bone loss was gradually increased and peaked at 6 weeks after CFA application (Fig. 1J). The volume of bone loss measured by 3D micro-CT imaging analysis software was significantly greater in CFA-treated rats compared with vehicle-treated rats from 3 weeks after CFA application (p<0.05, Fig. 1J).

### 2. Change in d-EMG activities following tooth pulp or periapical inflammation

**Figure 2:**
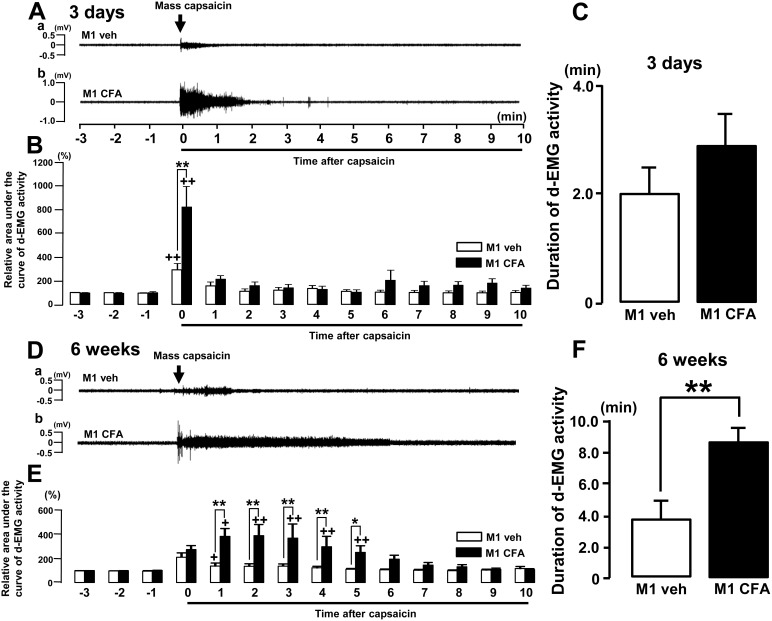
The d-EMG activities after tooth pulp or periapical inflammation. The capsaicin was applied into the Mass in the rats with CFA or vehicle application to the tooth pulp on day 3 and week 6. A: The d-EMG activity following capsaicin application into the Mass in the rats with pulpal-vehicle or pulpal-CFA application on day 3. B: Relative d-EMG activity following capsaicin application into the Mass in the rats with pulpal-vehicle or pulpal-CFA application. C: Mean duration of d-EMG activity following capsaicin application into the Mass in the rats with pulpal-vehicle or pulpal-CFA application. D: The d-EMG activity following capsaicin application into the Mass in the rats with pulpal-vehicle or pulpal-CFA application on week 6. E: Relative d-EMG activity following capsaicin application into the Mass in the rats with pulpal-vehicle or pulpal-CFA application. F: Mean duration of d-EMG activity following capsaicin application into the Mass in the rats with pulpal-vehicle or pulpal-CFA application. +: vs. −1 min, *: veh vs. CFA, +, *: p<0.05, ++, **: p<0.01.

We observed significant increase in the d-EMG activity within 1 min after capsaicin application into the Mass in pulpal CFA-applied rats (p<0.01, Fig. 2A, B). On the other hand, the duration of d-EMG activities after pulpal-CFA or pulpal-vehicle application was about 2 minutes, and no significant difference was observed between CFA and vehicle groups (Fig. 2C).

The d-EMG activities elicited by capsaicin application into the Mass at 6 weeks after pulpal-CFA application were shown in Fig. 2D. Compared with the pulpal-vehicle applied rats, the d-EMG activity was significantly increased (Fig. 2E) and the duration of the d-EMG activity was significantly lasted (Fig. 2F) after capsaicin application into the Mass at 6 weeks after pulpal CFA application.

### 3. pERK-IR and Fos-IR cells and Rostral-caudal distribution in Vi/Vc or Vc

**Figure 3:**
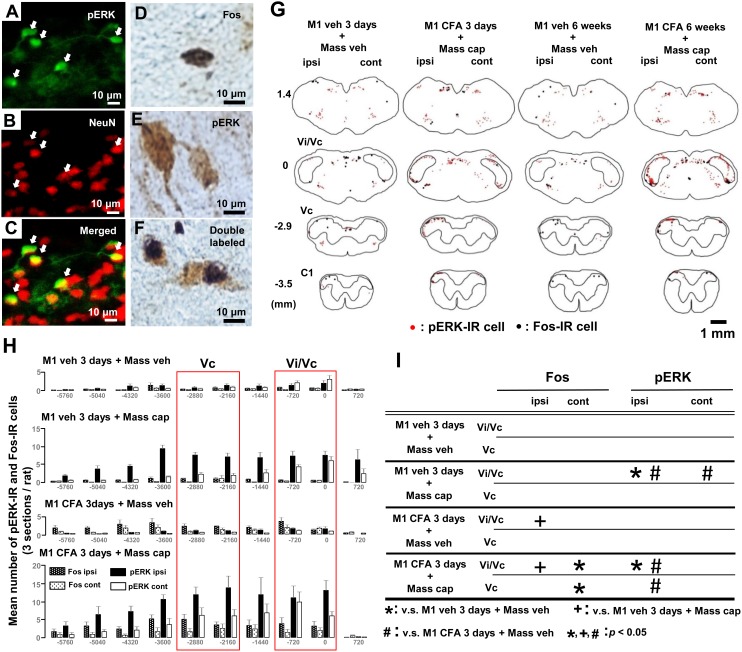
Photomicrographs and Rostral-caudal distribution for pERK-IR cells and Fos-IR cells after tooth pulp inflammation in Vi/Vc and Vc. A: pERK-IR cells. B: NeuN-IR cells. C: A merged with B. D: Fos-IR cell stained with nickel-conjugated DAB. E: pERK-IR cell stained with DAB. F: double stained cells (black: Fos-IR, brown: pERK-IR). G: Neurolucida drawings of pERK-IR (red and triangle dots) and Fos-IR cells (black and round dots) in the Vi/Vc and Vc. H: Rostral-caudal distribution of pERK-IR and Fos-IR cells in the Vi/Vc and Vc. I: *, + and # signs indicate significant expression of pERK-IR or Fos-IR cells compared to each region in M1 veh/Mass veh, M1 veh/Mass cap and M1 CFA/Mass veh on day 3, respectively. Arrows in A indicate pERK-IR cells, those in B indicate NeuN-IR cells and those in C are pERK-IR cells merged with NeuN-IR cells. The negative numbers on the left in G indicate the distance caudal to the obex (0 mm). cap: capsaicin, veh: vehicle, ipsi: ipsilateral, cont: contralateral.

**Figure 4:**
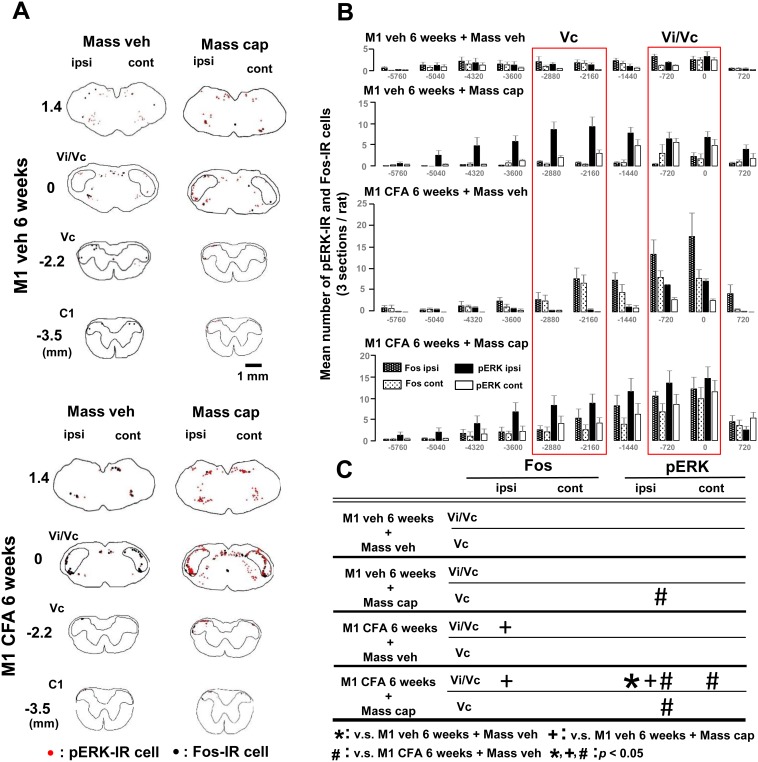
Rostral-caudal distribution for pERK-IR cells and Fos-IR cells after periapical inflammation in Vi/Vc and Vc. The capsaicin was applied into the Mass in the rats with CFA or vehicle application to the tooth pulp on week 6. A and B: Neurolucida drawings (A) and Rostral-caudal distribution (B) of pERK-IR and Fos-IR cells in the Vi/Vc and Vc. C: *, + and # signs indicate significant expression of pERK-IR or Fos-IR cells compared to each region in M1 veh/Mass veh, M1 veh/Mass cap and M1 CFA/Mass veh on day 3, respectively.

To examine the effect of M1 pulpitis or periapical periodontitis on the neuronal excitability, the ERK phosphorylation and Fos expression were studied in Vi/Vc and Vc neurons following unilateral capsaicin application into the Mass. A number of pERK-IR cells in the Vi/Vc and Vc induced by capsaicin at 3 days or 6 weeks after pulpal CFA application were double-stained with NeuN, a neuronal marker (Fig. 3A–C). However, some of the pERK-positive cells lacked NeuN staining. These results suggest that ERK phosphorylation occurs in grail cells as well as neurons. A large number of pERK-IR and Fos-IR cells were observed in the ventral portion of the Vi/Vc and in the dorsal portion of the Vc after capsaicin application into the Mass on day 3 or week 6 after CFA application into the M1 tooth pulp (Fig. 3G and 4A). Though we observed a few cells double-stained (Fig. 3F) with Fos (Fig. 3D) and pERK (Fig. 3E) antibodies, there are no double-stained cells in the Vi/Vc and Vc.

Rostral-caudal distribution of pERK-IR and Fos-IR cells in the medulla and upper cervical spinal cord were shown in Fig. 3G, H and Fig. 4A and B. A large number of pERK-IR and Fos-IR cells were observed in the Vi/Vc and Vc following capsaicin application into the ipsilateral Mass on day 3 or week 6 after pulpal CFA application (Fig. 3H and 4B). The total number of cells in Vc and Vi/Vc region indicated by red squares in Fig. 3H and 4B were calculated and the results of statistical analysis were shown in Fig. 3I and Fig. 4C, respectively. Blank indicate a lack of significant difference. The total number of pERK- or Fos-IR cells in the Vi/Vc or Vc in each group was compared respectively (Fig. 3I and Fig. 4C).

On day 3 (Fig. 3I), the number of Fos-IR cells was significantly larger in M1 CFA/Mass veh and M1 CFA/Mass cap rats compared to M1 veh/Mass cap rats in the ipsilateral Vi/Vc. The number of Fos-IR cells was significantly larger in M1 CFA/Mass capsaicin rats compared to M1 veh/Mass veh rats in the contralateral Vi/Vc and Vc.

Furthermore, the numbers of pERK-IR cells was significantly larger in M1 veh/Mass cap and M1 CFA/Mass cap rats compared to M1 veh/Mass veh rats and M1 CFA/Mass veh in the ipsilateral Vi/Vc or Vc, respectively. In the contralateral side to injections, the number of pERK-IR cells was significantly larger in M1 veh/Mass cap compared to M1 CFA/Mass veh in the Vi/Vc.

On week 6 (Fig. 4C), the number of Fos-IR cells was significantly larger in M1 CFA/Mass veh or M1 CFA/Mass cap rats compared to M1 veh/Mass cap rats in the ipsilateral Vi/Vc, respectively. The number of pERK-IR cells was also significantly larger in M1 CFA/Mass cap rats compared to M1 veh/Mass veh, M1 veh/Mass cap or M1 CFA/Mass veh rats in the ipsilateral Vi/Vc. Furthermore, the number of pERK-IR cells was significantly larger in M1 veh/Mass cap or M1 CFA/Mass cap rats compared to M1 CFA/Mass veh rats in the ipsilateral Vc. In the contralateral side to M1 injections, the number of pERK-IR cells was significantly larger in M1 CFA/Mass cap compared to M1 CFA/Mass veh in the Vi/Vc.

### 4. Comparison of pERK-IR and Fos-IR cells on day 3 and week 6

To examine the difference for the expression of pERK-IR and Fos-IR cells on day 3 and week 6, the ERK phosphorylation and Fos expression were studied in Vi/Vc and Vc neurons following unilateral saline or capsaicin application into the Mass.

In the rats with saline application into the Mass, the number of Fos-IR cells was significantly larger in M1 veh/Mass veh rats in the ipsilateral Vi/Vc or in M1 CFA/Mass veh rats in the ipsi- and contralateral Vi/Vc and ipsilateral Vc on week 6 compared to those in M1 veh/Mass veh rats on day 3 (p<0.05). The number of pERK-IR cells was also significantly larger in M1 CFA/Mass veh rats on week 6 compared to M1 veh/Mass veh on day 3, M1 veh/Mass veh on week 6 or M1 CFA/Mass veh rats on day 3 in the ipsilateral Vi/Vc (p<0.05).

In the rats with capsaicin application into the Mass, the number of Fos-IR cells was significantly larger in M1 CFA/Mass cap rats on week 6 compared to M1 veh/Mass cap rats on day 3 or M1 veh/Mass cap rats on week 6 in the ipsilateral Vi/Vc (p<0.05).

### 5. The effect of PD98059 in the Vi/Vc and Vc neurons

**Figure 5:**
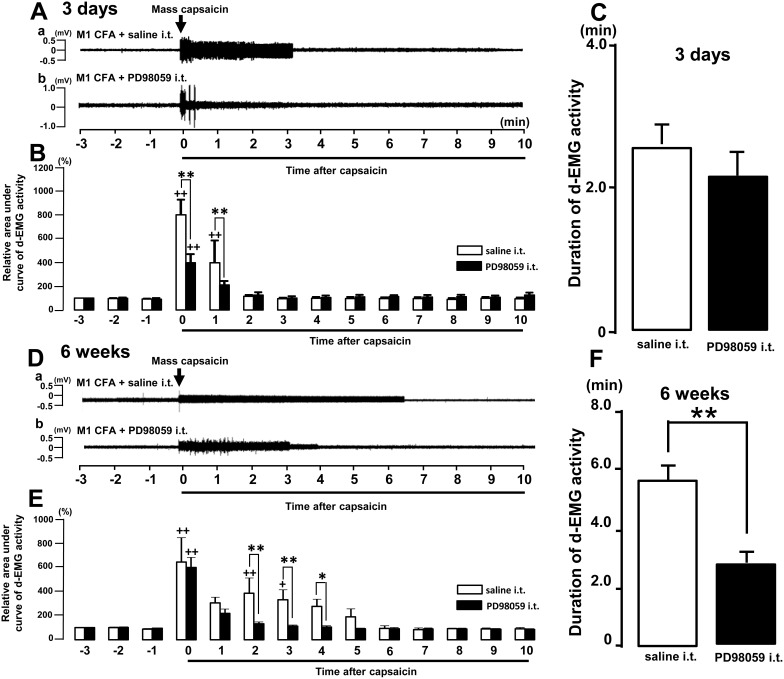
The effect of PD98059 on the d-EMG activities after tooth pulp or periapical inflammation. The capsaicin was applied into the Mass after pretreatment of PD98059 in the rats with M1 CFA application on day 3 and week 6. A: The d-EMG activity following capsaicin application into the Mass after pretreatment of PD98059 or saline in the rats with M1 CFA application on day 3. B: Relative d-EMG activity following capsaicin application into the Mass after pretreatment with PD98059 or saline in the rats with M1 CFA application. C: Mean duration of d-EMG activity following capsaicin application into the Mass after pretreatment with PD98059 or saline in the rats with M1 CFA application. D: The d-EMG activity following capsaicin application into the Mass after pretreatment of PD98059 or saline in the rats with M1 CFA application on week 6. E: Relative d-EMG activity following capsaicin application into the Mass after pretreatment with PD98059 or saline in the rats with M1 CFA application. C: Mean duration of d-EMG activity following capsaicin application into the Mass after pretreatment of PD98059 or saline in the rats with M1 CFA application. +: vs. −1 min, *: saline i.t. vs. PD98059 i.t., +,*: p<0.05., ++, **: p<0.01.

We further examined the effect of MEK1 inhibitor PD98059-pretreatment for the Mass hypersensitivity in the rats with M1 pulpitis or periapical periodontitis. We observed significant decrease in the d-EMG activity within 1 minute after capsaicin application into the Mass in the PD98059 i.t.-pretreated rats compared with saline i.t.-pretreated rats on day 3 after M1 CFA application (p<0.01, Fig. 5A, B).

On the other hand, the durations of d-EMG activities after capsaicin application were about 2 minutes, and no significant difference was observed between PD98059- or saline-pretreated rats on day 3 after M1 CFA application (Fig. 5C).

We observed significant attenuation in the d-EMG activity with in 2 minute after capsaicin application into the Mass in the PD98059 i.t.-pretreated rats compared with saline i.t.-pretreated rats on week 6 after M1 CFA application (p<0.01, Fig. 5D, E).

Furthermore, the mean duration of d-EMG activities after capsaicin application was significantly decreased in the PD98059-pretreated rats compared with saline-pretreated rats on 6 week after M1 CFA application (p<0.01, Fig. 5F).

**Figure 6:**
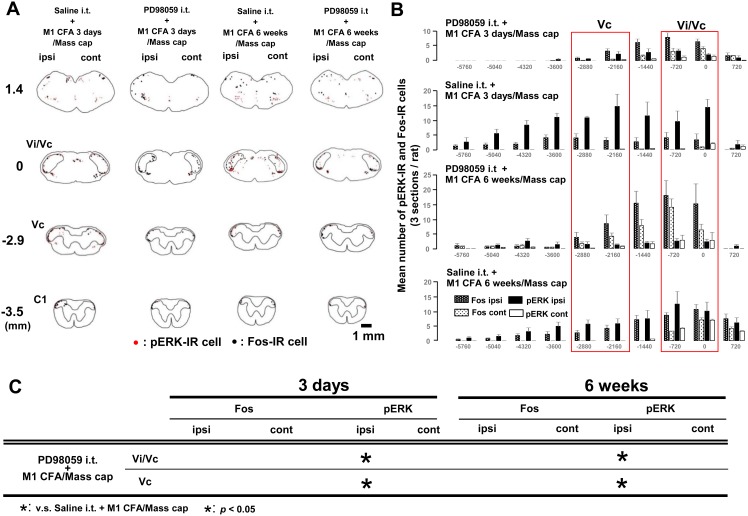
The effect of PD98059 on ERK phosphorylation in Vi/Vc and Vc neurons after tooth pulp or periapical inflammation. The capsaicin was applied into the Mass after PD98059 i.t. administration in the rats with M1 CFA on day 3 or week 6. A and B: Neurolucida drawings (A) and Rostral-caudal distribution (B) of pERK-IR and Fos-IR cells in the Vi/Vc and Vc. C: * indicates significant decrease in the number of pERK-IR or Fos-IR cells. Each region in each model was compared with M1 CFA/Mass cap rats with saline i.t. on day 3 or week 6, respectively.

To examine the effect of PD98059 on ERK phosphorylation caused by M1 pulpitis or periapical periodontitis, the change in the number of ERK-IR cells following pretreatment with PD98059 was studied in Vi/Vc and Vc following capsaicin application into the Mass (Fig. 6A and B).

On day 3 and week 6, the number of pERK-IR cells after Mass-capsaicin application was significantly decreased following PD98059 i.t. administration compared to saline i.t. administration in the ipsilateral Vi/Vc and Vc of the M1 CFA rats. Furthermore, the number of Fos-IR cells was not altered after the pretreatment of PD98059 or saline on day 3 or week 6 in the rats with M1 CFA/Mass cap (Fig. 6C).

## Discussion

Periapical inflammation frequently causes referred pain in the orofacial cutaneous tissue and/or muscle as well as tooth pulp [22]. It is well known that inflammation within the tooth pulp often extends into the periapical tissues after pulpal inflammation, resulting in pulpal necrosis and periapical inflammation. At the early stage of periapical inflammation, severe persistent pain occurs in the apex region of the tooth [23]. Eventually, periapical pain could change its’ quality from an acute to a chronic state. Chronic periapical pain is dull and persistent, lasts for a long period, and also causes referred pain in the orofacial skin and/or muscles. The orofacial referred pain originated from the periapical inflammation is difficult to diagnose and treat [3]. Despite increasing necessity of the clarification of the pathogenesis for the odontogenic referred pain in the orofacial region, it is still unclear on the mechanisms underlying extraterritorial orofacial persistent pain associated with periapical inflammation. In the present study, therefore, we examined the excitability of the Vi/Vc and Vc neurons in a rat model of experimental pulpitis/periodontitis and its’ involvement in the enhancement of d-EMG activity.

### 1. Rat model of periapical inflammation

It is well known that periapical bone loss is observed during development of the periapical inflammation, suggesting that the periapical bone loss could be one of the reliable indicators for the periapical inflammation [24]. Thus, we analyzed the time-course change in the bone loss using X-ray micro-CT imaging to quantify the periapical inflammation. The advantage of this technique is that the size of bone loss can be measured in same rats throughout the experimental period. Periapical bone loss was detected at 3 weeks after pulpal application of CFA, and the largest bone loss was observed at 6 weeks after CFA application to the pulp, indicating that chronic phase characterized by lesion stabilization was commenced from 6 weeks after CFA application. The time-course of bone loss we observed in this study is similar to that of previous study [24], suggesting that this model can be used as a reliable animal model with experimental periodontitis following long lasting pulpal inflammation.

### 2. Changes in the Vi/Vc and Vc neuronal activities following pulpal or periapical inflammation

The d-EMG activity elicited by noxious stimulation is known as a good indicator of the noxious responses in the trigeminal system [25]. We observed significant increase in the d-EMG activity after capsaicin application into the Mass on day 3 and week 6 after pulpal-CFA or pulpal-vehicle application. Furthermore, the d-EMG activity elicited by Mass-capsaicin application was significantly higher in M1 CFA-applied rats compared with M1-vehicle applied rats. The number of Fos-IR cells in the Vi/Vc was significantly larger in M1 CFA applied rats compared with M1 vehicle-applied rats on day 3 and week 6. On the other hand, ERK phosphorylation in the Vi/Vc and/or Vc neurons significantly increased in Mass-capsaicin applied rats compared with Mass-vehicle applied rats both in pulpal CFA- or vehicle applied rats. These indicate that noxious stimulus to the Mass induces the enhancement of noxious responses in the orofacial region following pulpal or periapical inflammation. It is strongly suggested that the increase in d-EMG activities and pERK expression in Vi/Vc and Vc neurons are the result of enhancement of Vi/Vc and/or Vc neuronal activities following pulpal or periapical inflammation. Taken together, the EMG activities and pERK expression were significantly enhanced after capsaicin application into the Mass on day 3 and week 6 after pulpal CFA application, suggesting that pathogenesis of pulpal or periapical inflammation might induce pain hypersensitivity in remote organs or tissues in the orofacial region.

We also observed that the duration of EMG activity was significantly longer at 6 weeks after pulpal CFA application compared with that of pulpal vehicle application. The enhancement of EMG activity and the increased duration were significantly reversed following i.t. administration of PD98059. These results indicate that periapical inflammation may induce and maintain pain hypersensitivity in the remote organs or tissues in orofacial region, whereas pulpal inflammation may only induce pain hypersensitivity. Furthermore, the ERK phosphorylation in Vi/Vc and Vc neurons has a pivotal role in the maintenance and/or modulation of pain processing in remote regions after tooth pulp or periapical inflammation.

### 3. Involvement of the Vi/Vc and Vc neurons in Mass hypersensitivity

Fos protein expression in SDH neurons following noxious stimulation of the peripheral structures is known to be caused 1–2 hours and lasted 24 hours after noxious stimulation [26], and therefore Fos protein expression in SDH neurons is thought to be a good marker of the excitation of nociceptive neurons [27]. The ventral portion of the Vi/Vc is known to have a various functions in trigeminal nociception [28]. Many Vi/Vc neurons are known to express Fos-IR products following CFA application into the Mass or noxious stimulation of the cornea [29], [30]. Many of them were also observed on both sides of the Vi/Vc following unilateral noxious stimulation.

It has been reported that the number of pERK-IR cells increases unilaterally in the Vc following increase in noxious stimulus intensity [31]. In the trigeminal system, ERK can be phosphorylated in a large number of dorsal horn neurons in the Vc and C1-C2 within 5 min after noxious stimulation to various orofacial regions; these pERK-IR neurons are somatotopically organized in Vc and C1-C2, and the number of pERK-IR neurons increases following increases in the noxious stimulus intensity [12], [20], indicating that ERK phosphorylation in Vc and C1-C2 neurons is a reliable marker of excitable neurons following orofacial noxious stimulation.

We observed that pERK expression was significantly enhanced after capsaicin application into the Mass both in M1 CFA- and vehicle-applied rats compared with Mass-vehicle applied rats in both Vi/Vc and Vc on day 3 and week 6. Furthermore, on day 3 after pulpal application, we observed a large number of Fos-IR cells in the bilateral Vi/Vc in pulpal CFA-applied rats. Meanwhile, on week 6 after pulpal-CFA application, we observed a large number of Fos-IR cells just in the ipsilateral Vi/Vc in pulpal CFA-applied rats, and the number of them was also significantly larger in CFA-applied rats compared with vehicle-applied rats. These findings suggest that nociceptive neurons in the Vi/Vc and Vc may have important roles for orofacial pain hypersensitivity associated with tooth pulp inflammation and periapical inflammation.

Furthermore, we could not observe significant change in the number of Fos-IR cells in these two areas between Mass-capsaicin and vehicle applied rats, suggesting that Fos expression may be mainly due to the CFA application to the tooth pulp but not Mass-capsaicin application.

## Conclusions

The present findings revealed that the Vi/Vc and Vc neurons was involved in the enhancement of Mass nociception under acute pulpal-inflamed state as well as chronic periapical inflammation, suggesting that nociceptive neurons in these areas are involved in pain hypersensitivity for orofacial muscle associated with acute pulpal and chronic periapical inflammation.
